# Multiple Mild Traumatic Brain Injuries Are Associated with Increased Rates of Health Symptoms and Gulf War Illness in a Cohort of 1990–1991 Gulf War Veterans

**DOI:** 10.3390/brainsci7070079

**Published:** 2017-07-09

**Authors:** Megan K. Yee, Patricia A. Janulewicz, Daniel R. Seichepine, Kimberly A. Sullivan, Susan P. Proctor, Maxine H. Krengel

**Affiliations:** 1 Boston VA Research Institute, Inc., Boston, MA 02130, USA; meganyee@bu.edu; 2 Department of Environmental Health, School of Public Health, Boston University, Boston, MA 02118, USA; paj@bu.edu (P.A.J.); tty@bu.edu (K.A.S.); susan.p.proctor.civ@mail.mil (S.P.P.); 3 Neuropsychology Laboratory, University of New Hampshire, Manchester, Manchester, NH 03101, USA; daniel.seichepine@unh.edu; 4 U.S. Army Research Institute of Environmental Medicine, Military Performance Division, Natick, MA 01760, USA; 5 VA Boston Healthcare System, Research Service, Boston, MA 02130, USA; 6 Department of Neurology, Boston University School of Medicine, Boston, MA 02118, USA

**Keywords:** mild traumatic brain injury, Gulf War Illness (GWI), chronic multisymptom illness, Gulf War

## Abstract

Recent research demonstrated a relation between traumatic brain injury (TBI), health symptoms and diagnosis of Gulf War Illness (GWI) in Gulf War Veterans, but no study has examined the impact of multiple mild TBIs (mTBIs). A total of 229 male Gulf War Veterans from the Ft Devens Cohort were categorized by a number of mTBIs reported. One-way ANOVA and chi-square test of independence were used to test for differences in total reported health symptoms and diagnosis of chronic multisymptom illness (CMI) or Kansas GWI criteria, two of the most common case definitions of GWI. A total of 72 veterans reported no mTBIs (31.4%), 26 reported one mTBI (11.4%), 25 reported two mTBIs (10.9%), and 106 veterans reported sustaining three or more mTBIs (46.3%). Veterans reporting two or more mTBIs (*p* < 0.01) or three or more mTBIs (*p* < 0.001) endorsed significantly higher rates of health symptoms than Veterans reporting no mTBIs. Significantly higher rates of CMI (*p* = 0.035) and Kansas GWI criteria (*p* < 0.001) were seen in the three or more mTBI group. Results suggest two mTBIs increase risk of health symptoms, but three mTBIs may be the threshold needed to sustain chronic symptom reporting needed for a formal diagnosis. These findings highlight the importance of implementing policies and procedures monitoring head injuries in military personnel.

## 1. Introduction

Veterans from the 1990–1991 Gulf War have consistently reported health symptoms since returning from the Middle East almost three decades ago [1,2,3,4,5,6,7,8,9,10,11,12,13]. Hallmark symptoms of the syndrome, commonly known as Gulf War Illness (GWI), include fatigue, musculoskeletal pain, respiratory problems, skin problems or rashes, gastrointestinal difficulties, changes in mood, and cognitive difficulties. Currently, etiological factors leading to GWI remain unclear, but common hypotheses include neurotoxicant exposures while in theatre, such as prophylactic treatments (e.g., pyridostigmine bromide pills) and environmental exposures (e.g., pesticides, sarin gas) [14,15,16,17]. The diverse range of symptoms and unclear etiological factors has made the diagnosis of GWI difficult. As a result, multiple diagnostic criteria have been developed. Two of the most commonly used criteria include Chronic Multisymptom Illness (CMI) developed by the Centers for Disease Control and Prevention and the Kansas GWI criteria [18,19,20]. Recent research demonstrated a relation between traumatic brain injury (TBI), health symptoms and rates of CMI in GW Veterans, which had not been previously examined [21]. However, to our knowledge, no study has examined the impact of multiple mTBIs on health symptoms or diagnosis of GWI in Veterans from the 1990–1991 Gulf War.

TBIs and mild TBIs (mTBIs), are of increasing concern, especially in cohorts where individuals are more likely to sustain multiple head injuries, such as sports communities and Veteran populations [22]. Mild TBI, also known as a concussion, occurs when an impact to the head or body results in one or more of the following: loss of consciousness (LOC) less than 30 minutes, inability to remember events immediately before or after the injury, alteration of consciousness (e.g., dazed, confused, disoriented), post-traumatic amnesia less than 24 hours, or neurological deficits [23]. Over the last decade, researchers have put-forth an overwhelming amount of evidence demonstrating significant chronic negative effects of repetitive mTBIs in individuals participating in contact sports, such as football [24,25]. Simultaneously, research on the impact of mTBIs in military populations has increased due to the use of improvised explosive devices (IEDs) in Operation Enduring and Iraqi Freedom (OEF/OIF).

A post-deployment survey study of OEF/OIF Veterans found that approximately 17% reported sustaining a mTBI in theater, with 59% of those reporting more than one mTBI. Veterans reporting mTBIs were at a higher risk for experiencing health symptoms, including headaches, cognitive problems, chest pain and gastrointestinal problems. Furthermore, veterans reporting more than one mTBI had an increased risk of experiencing health symptoms, such as headaches and sleep disturbances, compared to those only reporting a single head injury [26]. Studies specifically addressing the effects of multiple head injuries in military populations have found higher rates of post-concussive symptoms and sleep disturbances in Soldiers reporting multiple TBIs [27,28].

Recent research from the Ft. Devens Cohort Study demonstrated an association between self-reported TBI, chronic health symptoms and rates of CMI in Gulf War Veterans [21]. Veterans, surveyed in 1997–1998, self-reporting a TBI were more likely to meet CMI criteria and reported higher rates of chronic health symptoms than those who did not report a TBI. However, the study was limited due to a lack of information on the number and severity of TBIs experienced. The study also only examined rates of CMI, which has broad diagnostic criteria. Alternatively, the Kansas GWI criteria are more stringent. Recently, the Institute of Medicine (IOM) concluded CMI criteria should be used in clinical evaluations and Kansas GWI criteria should be used in research studies [29]. Comprehensive research should include diagnosis based on both case definitions. Therefore, the purpose of the current study was to determine whether GW Veterans reporting mTBIs were also reporting higher rates of chronic health symptoms and were more likely to meet CMI and/or Kansas GWI criteria. It was hypothesized that Veterans reporting more mTBIs would be more likely to report higher rates of health symptoms and more likely to meet CMI and/or Kansas GWI criteria.

## 2. Materials and Methods

### 2.1. Participants

Participants included 229 male Gulf War Veterans from the Ft. Devens Cohort Study, which is composed of Veterans who returned from war in 1991 through Ft Devens, Massachusetts. Initial assessments collected in 1991 and 1992–1993 were designed to assess psychological re-adjustment post-deployment. Later assessments collected in 1997–1998 and 2012–2014 included physical and emotional health concerns [3,4,6,7,9,10]. Review board approvals were obtained from the appropriate institutions prior to initiating survey distribution. Cross-sectional data collected from the 2012–2014 survey were analyzed for this study.

### 2.2. Self-Report of TBI

The following description of mTBI was provided:

“Some people have the misconception that mild traumatic brain injury (also known as ‘concussion’) only happens when you lose consciousness after being hit on the head or when the symptoms last for a long time. However, a mild traumatic brain injury occurs anytime you have an impact to the head that causes symptoms for any amount of time (e.g., seconds or longer). These symptoms include: sensitivity to light or noise, headache, dizziness, balance problems, nausea, vomiting, trouble sleeping, fatigue, confusion, difficulty remembering, difficulty concentrating, or loss of consciousness.”

Veterans were asked to self-report the number of head injuries sustained and total number of reported head injuries were summed and Veterans were categorized into one of four groups: no mTBIs, one mTBI, two mTBIs, and three or more mTBIs.

### 2.3. Health Symptom Checklist

The health symptom checklist is a 34-item self-report questionnaire assessing the presence and absence of bothersome health symptoms over the past 30 days. The symptoms spanned a range of body systems (e.g., cardiac, dermatological, gastrointestinal, genitourinary, musculoskeletal, neurological, neuropsychological, psychological, and pulmonary). Veterans were instructed to check “yes” if the symptom had been present or “no” if it had been absent, during the past 30 days. Number of self-reported health symptoms was summed for each Veteran.

### 2.4. Chronic Multisymptom Illness Criteria

CMI criteria, as defined by the Centers for Disease Control, include the presence of persistent health symptoms for at least 6 months in 2 of the following 3 categories: fatigue, musculoskeletal factors, and mood and/or cognition [18]. Veterans completed a questionnaire in which they self-reported the presence or absence of symptoms in each domain over the past 6 months. Based on their responses, Veterans were classified as meeting CMI criteria or not meeting CMI criteria. Six veterans, 2 in the no mTBI group, 2 in the one mTBI group, 1 in the two mTBI group, and 1 in the three mTBI group, did not have sufficient information to assess CMI status and were excluded from the analysis.

### 2.5. Kansas Gulf War Illness Criteria

Kansas GWI criteria include the presence of moderate to severe health symptoms that began during or after the Gulf War in 3 of the following 6 categories: fatigue, pain, neurological and/or cognitive and/or mood, skin, gastrointestinal, and respiratory [20]. Exclusion criteria include the diagnosis of a serious medical or psychiatric condition that could account for symptoms or influence accurate symptom reporting. Veterans completed a questionnaire in which they self-reported the presence and severity of symptoms, and indicated whether the symptom occurred before, during or after deployment and were classified accordingly as meeting Kansas GWI criteria or not.

### 2.6. Statistical Analysis

Due to the non-normal distribution of reported total number of mTBIs, a Spearman correlation was used to assess the relation between total number of self-reported health symptoms and total number of self-reported mTBIs. A one-way ANOVA was performed to test for a difference in mean health symptoms endorsed among the four mTBI groups. Post-hoc analyses utilized Tukey’s Honest Significant Difference test to determine which specific mTBI groups were significantly different. The chi-square test of independence or Fisher’s exact test if expected cell counts were less than 5 was performed to test for differences in CMI or Kansas GWI criteria rates. Standardized residuals adjusted for multiplicity using the Bonferroni method were used to determine which mTBI group was contributing to a significant result. While multiplicity was accounted for within each analysis, it was not accounted for across each analysis. Therefore, an alpha level of 0.05 was adopted for each individual analysis. Due to the wide range of total number of mTBIs reported, additional sensitivity analyses were conducted restricting the upper range of mTBIs to the third quartile to ensure outliers were not overly influencing the results.

## 3. Results

### 3.1. Participant Characteristics

Seventy-two veterans reported no mTBIs (31.4%), 26 reported one mTBI (11.4%), 25 reported two mTBIs (10.9%), and 106 veterans reported sustaining three or more mTBIs (46.3%). There were no significant differences between groups with respect to age, education and race (Table 1).

### 3.2. Correlation between Total Self-Reported Head Injuries and Health Symptoms

Overall, veterans reported a median of two mTBIs (IQR: 0–5) with a range 0 to 75 and a mean of 15.3 (sd = 8.7) health symptoms. Total self-reported mTBIs was significantly positively correlated with total self-reported health symptoms (rho = 0.417, *p* < 0.0001), indicating Veterans reporting more mTBIs also reported more health symptoms.

### 3.3. Health Symptom Checklist

**Figure 1:**
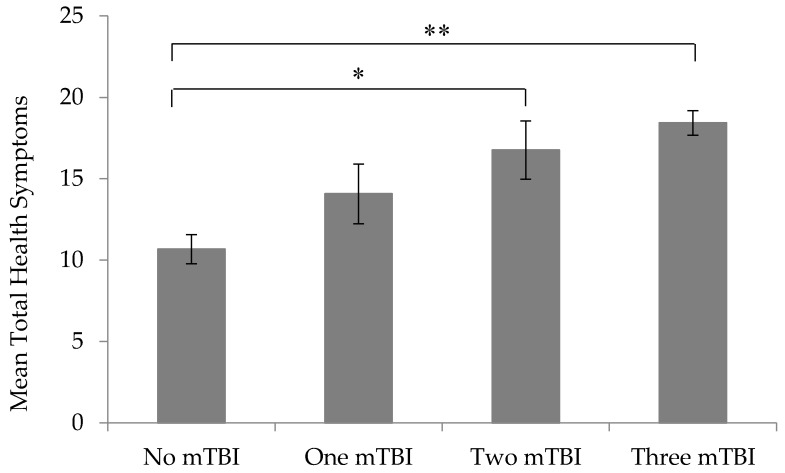
Mean total health symptoms by mild traumatic brain injury (mTBI) group. Error bars represent standard error. * *p* = 0.007. ** *p* < 0.001.

Veterans reported an average of 10.7 (sd = 7.5) health symptoms in the no mTBI group, 14.1 (sd = 9.4) health symptoms in the one mTBI group, 16.8 (sd = 9.0) health symptoms in the two mTBI group, and 18.4 (sd = 7.7) in the three or more mTBI group. The overall one-way ANOVA was significant (F = 13.93, *p* < 0.0001) indicating at least one of the mTBI groups reported a significantly different number of health symptoms. Post-hoc analysis revealed that Veterans with two mTBIs reported significantly more health symptoms than Veterans reporting no mTBIs (mean difference = 6.1, *p* = 0.007). Similarly, Veterans reporting three or more mTBIs reported significantly more health symptoms than Veterans reporting no mTBIs (mean difference = 7.8, *p* < 0.001). No significant differences were seen between the remaining mTBI groups (*p* > 0.05) (Figure 1). A sensitivity analysis restricting the range of mTBIs to 5 (which only affects the three or more mTBI group) still revealed a significant model (F = 7.55, *p* < 0.0001). Veterans reporting three or more mTBIs still endorsed significantly more health symptoms than veterans not reporting a mTBI (mean difference = 6.3, *p* < 0.001).

### 3.4. Chronic Multisymptom Illness

**Figure 2:**
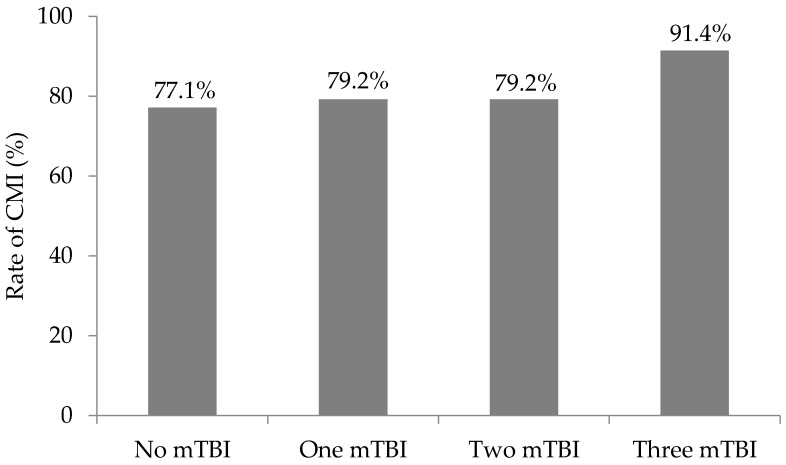
Rate of Chronic Multisymptom Illness by mild traumatic brain injury (mTBI) group. Six veterans are missing CMI status (2 in the no mTBI group, 2 in the one mTBI group, 1 in the two mTBI group, 1 in the three mTBI group). *p* = 0.035.

Overall, 84.3% of veterans met CMI criteria. Rate of CMI in the no mTBI group was 77.1% (*n* = 54), 79.2% (*n* = 19) in the one mTBI group, 79.2% (*n* = 19) in the two mTBI group, and 91.4% (*n* = 96) in the three or more mTBI group (Figure 2). Fisher’s exact test revealed a significant difference between the mTBI groups (*p* = 0.035). Adjusting for eight cells, the absolute value of a standardized residual greater than 2.7 (0.05/8 = 0.006, associated critical value of 2.7) indicates a particular cell is contributing to the significant result. The standardized residual for the three or more mTBI group meeting CMI criteria was 2.8 (−2.8 for the three or more mTBI group not meeting CMI criteria) indicating the rate of CMI in the three or more mTBI group was higher than expected. The standardized residuals for the remaining mTBI groups were less than 2.7. After restricting the range of mTBIs to 5 in a sensitivity analysis, 89.1% (*n* = 49) of the veterans in the three or more mTBI group met criteria for CMI. However, the significant difference between the mTBI groups no longer remained (*p* > 0.05).

### 3.5. Kansas Gulf War Illness

**Figure 3:**
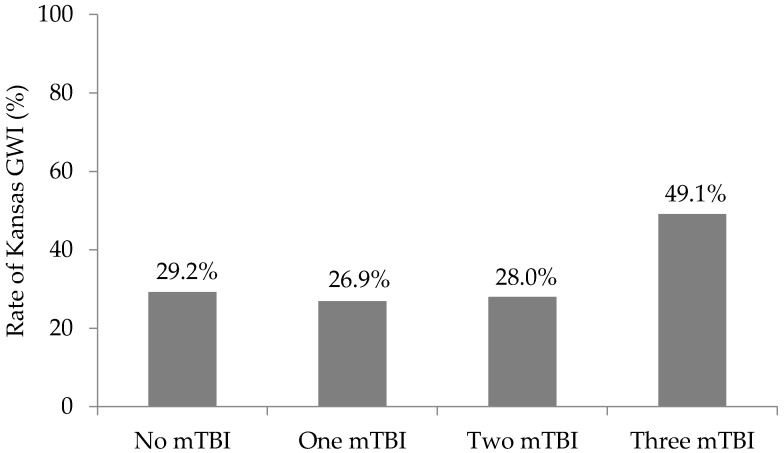
Rate of Kansas Gulf War Illness (GWI) criteria by mild traumatic brain injury (mTBI) group. *p* = 0.016.

Overall, 38.0% of the veterans met Kansas GWI criteria. Rate of Kansas GWI criteria in the no mTBI group was 29.2% (*n* = 21), 26.9% (*n* = 7) in the one mTBI group, 28.0% (*n* = 7) in the two mTBI group, and 49.1% (*n* = 52) in the three or more mTBI group (Figure 3). The resulting chi-square test revealed a significant difference between the mTBI groups (*p* = 0.016). Adjusting for eight cells, the absolute value of a standardized residual greater than 2.7 (0.05/8 = 0.006, associated critical value of 2.7) indicates a particular cell is contributing to the significant result. The standardized residual for the three or more mTBI group meeting Kansas GWI criteria was 3.2 (−3.2 for the three or more mTBI group not meeting Kansas GWI criteria). This suggests that more Veterans in the three mTBI group met Kansas GWI criteria than expected. The standardized residuals for the remaining mTBI groups were less than 2.7. After restricting mTBIs to a maximum of 5 in the sensitivity analyses, 51.8% (*n* = 29) of the Veterans in the three or more mTBI group met Kansas GWI criteria. The chi-square test of independence still demonstrated a significant difference between mTBI groups (*p* = 0.027). Similar to the main results, the standardized residuals for the three or more mTBI group meeting Kansas GWI criteria was 3.0 (−3.0 for the three or more mTBI group not meeting Kansas GWI criteria) indicating veterans were meeting criteria more than expected.

## 4. Discussion

The current study examined the relation between the number of self-reported mTBIs and health symptoms in a cohort of 1990–1991 Gulf War Veterans. Though not previously considered to be a contributing factor, recent research revealed that Gulf War Veterans reporting a TBI also endorsed higher rates of health symptoms and were more likely to meet CMI criteria [21]. However, the study was limited due to a lack of information regarding number and severity of TBIs. The current study utilized follow-up data focusing exclusively on mTBIs in the same cohort of Gulf War Veterans. Overall, it was demonstrated that Gulf War Veterans reporting two or more mTBIs also endorsed higher rates of health symptoms compared to Veterans reporting no exposure to mTBIs. Additionally, Veterans in the three or more mTBI group meet both CMI and Kansas GWI criteria more than expected.

These results also coincide with research on multiple head injuries in other cohorts, such as sports communities, where individuals are at an increased risk for multiple mTBIs [22,24,25]. Within the last decade, research on repetitive mTBIs in football players has consistently demonstrated an association between multiple head injuries and chronic negative health effects. A prospective cohort study of collegiate football players across the United States found an association between repetitive concussions and increased symptom duration and slower recovery time [30]. Studies of retired professional football players have consistently associated head injuries with worse health, increased rates of depression and an increased risk for neurodegenerative disorders years after play [31,32,33].

Negative consequences of multiple mTBIs are not exclusively limited to high-risk cohorts, but are also generalizable to the other populations. Increased rates of health symptoms were found in a community sample of adults and children sustaining a recurrent TBI of any severity within one year of an initial head injury compared to matched controls with no recurrent TBI [34]. The Transforming Research and Clinical Knowledge study collected information on individuals requiring a computed tomography scan after sustaining a head injury. Investigators found that individuals with a history of at least one head injury were more likely to report hepatic, musculoskeletal, spinal, neurological, pulmonary, and ear, nose or throat conditions than individuals sustaining their first TBI. Individuals with a history of TBI also reported higher rates of anxiety, depression and sleep disorders. Six months post-injury, individuals with a TBI history had higher rates of somatic symptoms, depression, anxiety, and worse processing speed and verbal learning. Additionally, individuals with a history of TBI were less likely to have returned to work [35]. However, it should be noted that in this study only 82% of the index head injuries were mTBIs and history of head injury had to be accompanied by a loss of consciousness. Unsurprisingly, individuals with a history of TBI have been found to have lower life satisfaction one-year post-head injury compared to individuals with no such history [36].

Currently, the threshold for the number of mTBIs an individual can sustain before experiencing negative consequences is unclear. If a threshold exists, it could directly impact policy. The results of this study suggest that the threshold may be two mTBIs for chronic health symptoms, as Veterans in the two mTBI and in the three or more mTBI group endorsed significantly more health symptoms than Veterans with no mTBIs. This is further strengthened by the finding that Veterans endorsing one mTBI reported similar rates of health symptoms as veterans with no mTBIs.

Our health symptom findings coincide with Miller and colleagues [27] research, which demonstrated that active duty Soldiers with two or more head injuries (<3 months) reported more health symptoms. Alternatively, work from Dretsch and colleagues [28] in active duty Soldiers preparing for deployment found an association between three or more concussions and increased rates of post-concussive symptoms measured by the neurobehavioral symptom inventory, which coincides with our CMI and Kansas GWI criteria findings. However, Miller et al. [27] only grouped Soldiers by no, one, or two or more mTBIs. Therefore, it is possible that the Soldiers with three or more mTBIs were driving the higher rate of symptom endorsement, which would be more consistent with the work of Dretsch [28].

Differences between the current study and Dretsch et al. [28] may account for the different health symptoms findings. Dretsch [28] studied active duty Soldiers whose head injuries were most likely more recent than the current study, in which veterans most likely sustained their injuries when they were active duty over two decades ago. The average age of the active Soldiers in Dretsch et al. [28] 26 years old, was also much younger than the average age in the current study. The effects of multiple mTBIs on chronic health symptoms later in life may be different then the effects seen closer to time of injury.

Finally, Gulf War veterans are a unique population due to the unique neurotoxicant exposures they encountered while in theatre, including pesticides and nerve agents, which may compound the effect of head injuries. This is known as the multiple hit hypothesis, which suggests multiple insults to the nervous system can cause chronic neuroinflammation due to a persistent neuroimmune response [37,38,39].

Contrary to our health symptom results, our findings demonstrated that Veterans reporting three or more mTBIs were more likely to meet CMI or Kansas GWI criteria. The Kansas GWI criteria results were particularly striking, as the rate of diagnosis nearly doubled in the group of Veterans reporting at least three mTBIs compared to Veterans not reporting any mTBIs. However, Veterans endorsing two mTBIs did not endorse higher rates of CMI or Kansas GWI criteria. It is important to note that the CMI results were no longer significant in the sensitivity analysis when the number of mTBIs were reduced to 5. This may suggest that the initial result may have been influenced by a limited number of Veterans reporting a high number of mTBIs. However, the difference in the sensitivity analysis between the CMI and Kansas GWI illness may be a reflection of the diagnostic criteria themselves. CMI criteria are broad and may potentially over diagnose Veterans, as demonstrated by the high rate in this sample (84.3%). Kansas GWI criteria are more stringent (38.0% in this sample), but may exclude some Veterans with other comorbid conditions. While the health symptom results and the diagnostic criteria results in this study are seemingly contradictory at first, the difference in the results may suggest that while it may only take two mTBIs to increase rates of health symptoms, it may take at least three mTBIs before symptoms are severe enough for a Veteran to meet criteria for a formal diagnosis. However, this result needs to be interpreted with caution as more research is needed, especially regarding the timing of mTBIs in relation to symptom development, and other exposures which may contribute to (GWI).

While the current study has its strengths, there are some limitations inherent in the study design that should be addressed. Though veterans were provided with a mTBI definition that has been used in a multitude of published studies, only retrospective self-report data was used with no external verification of mTBIs. This may be particularly relevant in the current study, as Veterans are asked about events occurring over two decades prior. Timing of the mTBIs, whether they occurred before, during or after the war, was also not assessed. Further, if Veterans reported multiple mTBIs, the time between each mTBI was not collected. Both of these factors may alter the effect on health symptoms, and should be assessed in future studies. Similarly, health symptoms were self-reported with no clinician involvement. It is possible that some Veterans tended to endorse items indiscriminately or Veterans reporting more mTBIs were primed to report more health symptoms (e.g., response bias). Clinical evaluations for both mTBI exposure and symptom reporting may be beneficial to future studies.

To our knowledge, this is the first study to examine the association between multiple mTBIs, health symptoms and rate of CMI or Kansas GWI criteria in a cohort of Gulf War Veterans. Both subjective and objective evidence continues to support the seriousness of repetitive brain injuries in multiple diverse cohorts. However, the threshold for the number of head injuries that can be sustained before an increase in risk of chronic symptoms still remains unclear. The current study indicates that as few as two mTBIs may significantly increase the risk of chronic health symptoms, but three mTBIs may be the threshold needed to sustain chronic symptom reporting. These findings highlight the importance of implementing procedures and policies to closely monitor head injuries within the military.

## Figures and Tables

**Table 1:** Participant Characteristics by Mild Traumatic Brain Injury (mTBI) Group.

	No mTBI (*N* = 72)	One mTBI (*N* = 26)	Two mTBI (*N* = 25)	Three mTBI (*N* = 106)
Age, years	57.3 ± 9.0	57.2 ± 9.5	56.5 ± 7.7	54.4 ± 7.5
Education, years	14.5 ± 2.8	14.8 ± 2.7	13.8 ± 3.8	14.0 ± 2.7
% Caucasian	66 (91.7%)	26 (100.0%)	24 (96.0%)	95 (89.6%)

Note: No significant differences were seen between groups for participant characteristics.
